# Visible Light-Driven Direct Z-Scheme Ho_2_SmSbO_7_/YbDyBiNbO_7_ Heterojunction Photocatalyst for Efficient Degradation of Fenitrothion

**DOI:** 10.3390/molecules29245930

**Published:** 2024-12-16

**Authors:** Liang Hao, Jingfei Luan

**Affiliations:** 1 School of Physics, Changchun Normal University, Changchun 130032, China; hliang0725@163.com; 2 State Key Laboratory of Pollution Control and Resource Reuse, School of the Environment, Nanjing University, Nanjing 210093, China

**Keywords:** Ho_2_SmSbO_7_/YbDyBiNbO_7_ heterojunction, direct Z-scheme, fenitrothion, visible light absorption, photocatalytic efficiency, degradation pathway, degradation mechanism

## Abstract

A highly versatile Z-scheme heterostructure, Ho_2_SmSbO_7_/YbDyBiNbO_7_ (HYO), was synthesized using an ultrasonic-assisted solvent thermal method. The HYO heterojunction, composed of dual A_2_B_2_O_7_ compounds, exhibits superior separation of photogenerated carriers due to its efficient Z-scheme mechanism. The synergistic properties of Ho_2_SmSbO_7_ and YbDyBiNbO_7_, particularly the excellent visible light absorption, enable HYO to achieve exceptional photocatalytic performance in the degradation of fenitrothion (FNT). Specifically, HYO demonstrated an outstanding removal efficiency of 99.83% for FNT and a mineralization rate of 98.77% for total organic carbon (TOC) during the degradation process. Comparative analyses revealed that HYO significantly outperformed other photocatalysts, including Ho_2_SmSbO_7_, YbDyBiNbO_7_, and N-doped TiO_2_, achieving removal rates that were 1.10, 1.20, and 2.97 times higher for FNT, respectively. For TOC mineralization, HYO exhibited even greater enhancements, with rates 1.13, 1.26, and 3.37 times higher than those of the aforementioned catalysts. Additionally, the stability and durability of HYO were systematically evaluated, confirming its potential applicability in practical scenarios. Trapping experiments and electron paramagnetic resonance analyses were conducted to identify the active species generated by HYO, specifically hydroxyl radicals (•OH), superoxide anions (•O_2_^−^), and holes (h^+^). This facilitated a comprehensive understanding of the degradation mechanisms and pathways associated with FNT. In conclusion, this study represents a substantial contribution to the advancement of efficient Z-scheme heterostructure and offers critical insights for the development of sustainable remediation approaches aimed at mitigating FNT contamination.

## 1. Introduction

Over the past seven decades, organophosphate pesticides have been widely used and highly effective agrochemicals. Fenitrothion (FNT), a prominent representative within this category, has been in use since 1959 for controlling the growth of fruits, cotton, and cereals, as well as for pest management [1,2,3,4,5]. Despite its effectiveness, FNT is highly toxic as it functions by inhibiting acetylcholinesterase. Even trace amounts of FNT in aquatic environments could cause severe damage to the nervous system, sensory functions, visual systems, and cognitive abilities in both humans and animals [6,7,8]. Consequently, FNT has been banned in European countries, but it remains widely used in Russia, the United States, and other developing nations [5]. Hence, the development of an effective method for removing FNT from aquatic environments is of crucial importance.

Photocatalysis has developed into a promising, sustainable approach for mitigating water pollution, offering an efficient means to degrade contaminants in aquatic environments [9,10,11,12]. It utilizes photogenerated holes and electrons to activate H_2_O and O_2_, respectively, thereby producing reactive oxygen species capable of degrading toxic organic pollutants in wastewater [13,14]. Photocatalysts are the core of photocatalytic technology. Traditional single-metal oxide photocatalysts, which were among the earliest to be investigated, are limited to the utilization of UV or near-visible light [15,16,17]. Recent research efforts have been focused on overcoming these limitations by developing efficient visible light-responsive photocatalysts. Pyrochlore structure compound oxide photocatalysts with the A_2_B_2_O_7_ composition demonstrated remarkable thermal and chemical stability alongside abundant surface acid-base centers, strong responsiveness to visible light, significant oxygen vacancies, and superior oxygen ion conduction capabilities, thereby highlighting the potential for efficient visible light activation and effective degradation of various pollutants [18,19,20,21,22,23,24,25]. For instance, Zhang et al. achieved a photocatalytic removal efficiency (PRE) of 76.6% for methyl orange by utilizing La_2_Ce_2_O_7_ powder synthesized via the sol-gel method and subjected to visible light exposure (VE) for 300 min [26]. Similarly, Yao et al. developed a facile in situ precipitation method to synthesize Gd_2_YSbO_7_ nanophotocatalyst, which exhibited a notable PRE of 82.45% for benzotriazole under VE for 145 min [27]. Additionally, Devi et al. reported a PRE of 66% for methylene blue using YGdTi_2_O_7_ powder synthesized through the sol-gel method and exposed to visible light for 180 min [28].

Research has revealed the potential for enhancing the photocatalytic properties of Bi_2_InTaO_7_ by means of structural modification [29]. Previous research on ZnO and TiO_2_ has provided significant insights into the enhancement of photocatalytic activity through doping with various elements. For instance, Phuruangrat et al. synthesized Ho-doped ZnO and observed a notable increase in the photodegradation of methylene blue compared to pure ZnO [30]. Similarly, Ali et al. synthesized Sm-doped ZnO, which also exhibited a marked improvement in methylene blue degradation relative to pristine ZnO [31]. In another study, Nasser et al. reported that Sb-doped ZnO demonstrated enhanced degradation of Rhodamine B compared to undoped ZnO [32]. In the context of TiO_2_, Pérez et al. synthesized Yb-doped TiO_2_ and found significant improvements in the degradation of methylene blue over undoped TiO_2_ [33]. Radha et al. contributed further by synthesizing Dy-doped TiO_2_, which showed similar enhancements in the degradation of methylene blue and methyl orange compared to undoped TiO_2_ [34]. Furthermore, Sood et al. presented findings on Bi-doped TiO_2_, revealing notable improvements in the degradation of Alizarin red S [35], while Kong et al. synthesized Nb-doped TiO_2_ and demonstrated significant enhancements in the degradation of acetaldehyde compared to undoped TiO_2_ [36]. Collectively, these studies highlight the efficacy of incorporating elements such as Ho, Sm, and Sb to enhance the photocatalytic performance of ZnO, as well as Yb, Dy, Bi, and Nb to improve the photocatalytic efficacy of TiO_2_. Based on these findings, we hypothesize that novel single-crystal photocatalysts, namely Ho_2_SmSbO_7_ and YbDyBiNbO_7_, would display superior photocatalytic performance through the strategic substitution of elements within Bi_2_InTaO_7_.

However, single-component photocatalytic materials encounter challenges in practical applications, particularly the high rate of recombination among photogenerated carriers (PGCs) [37,38]. To resolve this matter, binary heterojunction photocatalytic materials have been put forward [39,40,41]. The internal electric field generated between the two components of a binary heterojunction could effectively promote the spatial separation of PGCs, leading to enhanced photocatalytic performance compared to single-component materials. For example, when high-quality type II heterojunctions were formed, the photodegradation performance of the fabricated samples could be significantly improved [42,43]. Lu et al. developed a type-II ZnIn_2_S_4_/BiPO_4_ heterojunction using a facile hydrothermal method. Under VE for 90 min, this material achieved an impressive tetracycline (40 mg/L) removal rate of 88%, significantly outperforming pure ZnIn_2_S_4_ and BiPO_4_ [44]. Similarly, Bhoi et al. synthesized a type-II Bi_2_S_3_/BiFeO_3_ heterostructure catalyst using a combustion method, which demonstrated a remarkable 96% PRE of carbendazim (10 mg/L) within 120 min under VE, greatly exceeding the performance of pure Bi_2_S_3_ and BiFeO_3_ [45].

In recent years, Z-type heterojunctions have gained prominence over traditional heterojunctions due to their ability to achieve efficient spatial separation of PGCs while maintaining higher priority redox potentials during photodegradation [46,47,48]. However, reports on A_2_B_2_O_7_-based Z-type heterojunction photocatalysts are limited to combinations of one A_2_B_2_O_7_ component with another semiconductor photocatalytic material. For instance, Zhu et al. prepared a direct Z-type Bi_2_Sn_2_O_7_/g-C_3_N_4_ heterojunction using an ultrasonic-assisted hydrothermal method [49], Huang et al. synthesized a direct Z-type Bi_2_Sn_2_O_7_/SnO_2_ heterostructure material via a one-pot hydrothermal method [50], and Zhu et al. fabricated a direct Z-type La_2_Ti_2_O_7_/Ag_3_PO_4_ heterojunction utilizing an in-situ precipitation method [51]. Nevertheless, few studies have explored direct Z-type photocatalysts composed of dual A_2_B_2_O_7_ components. Therefore, it is both necessary and timely to develop a direct Z-type photocatalyst with dual A_2_B_2_O_7_ components and explore its photocatalytic applications.

In light of this, guided by a suitable band structure design, we have synthesized a novel direct Z-type Ho_2_SmSbO_7_/YbDyBiNbO_7_ (HYO) heterojunction photocatalyst using an ultrasonic-assisted hydrothermal method. The investigation into the crystal structure, morphological traits, and optical properties of this photocatalyst was carried out in detail, validating the possible direct Z-type mechanism. The photocatalytic performance of the HYO composite system for FNT degradation under VE was evaluated, and its stability was verified through cyclic experiments. Additionally, the types of radicals involved in the FNT degradation process, the photocatalytic mechanism, and the degradation pathways were explored. This research underscores the efficacy and innovative qualities of the HYO heterojunction catalyst in the degradation of FNT, showcasing its potential as a viable material for environmental cleanup.

## 2. Results and Discussion

### 2.1. Characterization of Photocatalysts

**Figure 1:**
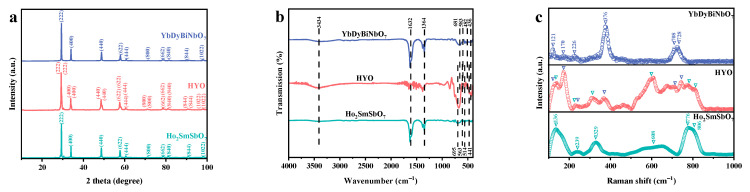
(**a**) XRD, (**b**) FTIR, and (**c**) Raman plots of Ho_2_SmSbO_7_, YbDyBiNbO_7_, and HYO.

The crystalline phase purity of the Ho_2_SmSbO_7_, YbDyBiNbO_7_, and HYO nanocomposites was assessed using X-ray diffraction (XRD), with the corresponding diffractograms presented in Figure 1a. Additionally, representative XRD patterns of Ho_2_SmSbO_7_ and YbDyBiNbO_7_ were compared with the reference standard card (PDF#53-1042) of Bi_2_InNbO_7_ in Figure S1. The reference material Bi_2_InNbO_7_ possessed the same space group and crystal structure as Ho_2_SmSbO_7_ and YbDyBiNbO_7_, denoting their similarity in the A_2_B_2_O_7_ compound class. The absence of other impurities confirmed the high purity of the synthesized catalysts. The well-defined diffraction peaks in all patterns indicated the excellent crystallinity of the catalysts. Pure Ho_2_SmSbO_7_ exhibited distinct peaks at 29.6°, 34.2°, 49.1°, 58.3°, 61.1°, 71.9°, 79.6°, 82.1°, and 91.9°, which could be attributed to the (222), (400), (440), (622), (444), (800), (662), (840), and (844) planes, respectively. Similarly, YbDyBiNbO_7_ showed prominent peaks at 29.4°, 34.0°, 48.8°, 57.9°, 60.8°, 71.5°, 79.1°, 81.5°, and 91.4°, corresponding to the same planes. The diffraction peaks observed in the HYO nanocomposite exhibited distinctive contributions from both Ho_2_SmSbO_7_ and YbDyBiNbO_7_, indicating the successful incorporation of both phases into the composite structure. The overlapping of diffraction peaks from Ho_2_SmSbO_7_ and YbDyBiNbO_7_ further substantiated the successful formation of the HYO composite. Importantly, no additional peaks corresponding to impurities were detected, confirming the chemical purity of the synthesized catalysts.

Further structural validation was carried out through Rietveld refinement of Ho_2_SmSbO_7_ and YbDyBiNbO_7_ using the Materials Studio program, and the refinement yielded Rp-values of 5.05% and 6.10%, respectively, indicating a good agreement between experimental and theoretical data. Figures S2a and S3a depict these results, validating the pyrochlore-type crystal structure of both compounds. Both materials crystallized in a cubic structure characterized by the Fd3m space group, confirming their purity as single-phase compounds. The crystal lattice dimensions were determined to be 10.531 Å for Ho_2_SmSbO_7_ and 10.742 Å for YbDyBiNbO_7_. The atomic structures of Ho_2_SmSbO_7_ and YbDyBiNbO_7_ were illustrated in Figures S2b and S3b, respectively, and were based on the parameters listed in Tables S1 and S2.

The compound Ho_2_SmSbO_7_ contained two varieties of Ho–O bonds: the longer bonds, denoted as Ho-O(1), measure 2.272 Å, while the shorter Ho–O(2) bonds were 2.280 Å in length. The differences in bond lengths were expected to induce distortions in the MO_6_ octahedra (M = Sm^3+^ and Sb^5+^), leading to a reduction in the recombination rate of PGCs and consequently enhancing photocatalytic activity [29,52]. The M–O–M bond angle was measured at 145.435°, with previous studies indicating that bond angles closer to 180° facilitate better PGC mobility and photocatalytic activity [29,52]. The larger M–O–M angle in Ho_2_SmSbO_7_ further contributed to its photocatalytic efficacy. Likewise, in YbDyBiNbO_7_, variations in the lengths of A–O(1) (A = Yb^3+^ and Dy^3+^) bonds measuring 2.618 Å and A-O(2) bonds measuring 2.278 Å resulted in distortions within the M’O_6_ octahedra (M’ = Bi^3+^ and Nb^5+^) [29,52]. The M’–O–M’ angle in YbDyBiNbO_7_ was determined to be 133.273°. These findings validate the structural stability of the samples and emphasize their capability as potent photocatalytic materials for use in environmental applications.

Further characterization of the functional groups in the prepared samples was conducted using Fourier Transform Infrared (FTIR) spectroscopy. The FTIR spectrum of the HYO nanocomposite, as depicted in Figure 1b, encompassed all the characteristic peaks observed in the individual components, Ho_2_SmSbO_7_ and YbDyBiNbO_7_. The absorption band observed at 441 cm^−1^ could be ascribed to the vibrational bending associated with Sb–O bonds [53], whereas the peak detected at 695 cm^−1^ was attributed to the vibrational bending of Sb–O–Sb bonds [54]. The tensile vibrations of Sm–O were observed at 514 cm^−1^ [55], and the vibrational bending of Ho–O was detected at 563 cm^−1^ [56]. The tensile vibrations of Dy–O were identified at 436 cm^−1^ [57], while the vibrational bending of Nb–O was detected at 681 cm^−1^ [58]. The absorption band observed at 482 cm^−1^ was attributed to the vibrational modes of Bi–O bonds within the distorted BiO_6_ octahedra [59], whereas the peak at 583 cm^−1^ corresponded to the tensile vibrations of Yb–O within the (YbO_6_) and (YbO_8_) complexes [60]. Additionally, the broad band centered at 3434 cm^−1^ corresponded to the tensile vibrations of hydroxyl groups, likely originating from adsorbed water or crystalline water [61]. The absorption band identified at 1632 cm^−1^ corresponded to the vibrational bending of the H–O–H structure, thereby indicating the presence of water [61]. In addition, the peaks observed in the vicinity of 1364 cm^−1^ were attributed to the symmetric distortion modes of C–H bonds [62].

Figure 1c presents the Raman spectrum of the synthesized samples, revealing several key features indicative of their structural characteristics. Notably, a peak at 136 cm^−1^ corresponded to the vibrational bending associated with the Sb–O–Sb bond [63]. A prominent peak at 239 cm^−1^ was attributed to the vibrational modes of Sm–O bonds [64], while tensile vibrations of Ho–O bonds were observed at 329 cm^−1^ [65]. Additional peaks appearing at 608 cm^−1^, 776 cm^−1^, and 806 cm^−1^ were assigned to Sb–O bond vibrations [66]. The peak at 121 cm^−1^ was linked to Bi^3+^ cations present within [BiO_3_] and [BiO_6_] units, whereas the peak at 170 cm^−1^ pertained to bismuth units featuring a bridging anion [67]. Furthermore, the peak at 226 cm^−1^ was ascribed to the bending modes of Nb–O–Nb [68], and the peak at 376 cm^−1^ corresponding to the A_g_ and F_g_ modes of Yb–O bonds [69]. Additionally, a peak at 708 cm^−1^ was associated with Dy–O bonds [70]. Collectively, the distinct peaks observed in the Raman spectrum of HYO included 121 cm^−1^, 136 cm^−1^, 170 cm^−1^, 226 cm^−1^, 239 cm^−1^, 329 cm^−1^, 376 cm^−1^, 608 cm^−1^, 708 cm^−1^, 776 cm^−1^, and 806 cm^−1^. Overall, the results obtained from FT-IR and Raman spectroscopic analyses, when considered alongside XRD data, provide compelling evidence for the successful formation of a heterostructure between Ho_2_SmSbO_7_ and YbDyBiNbO_7_.

**Figure 2:**
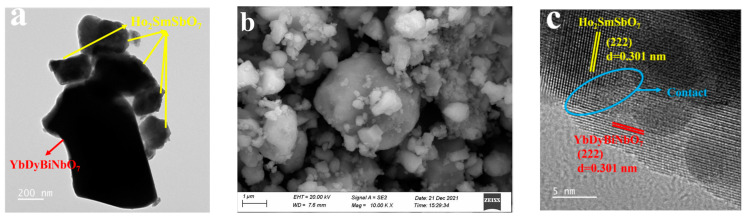

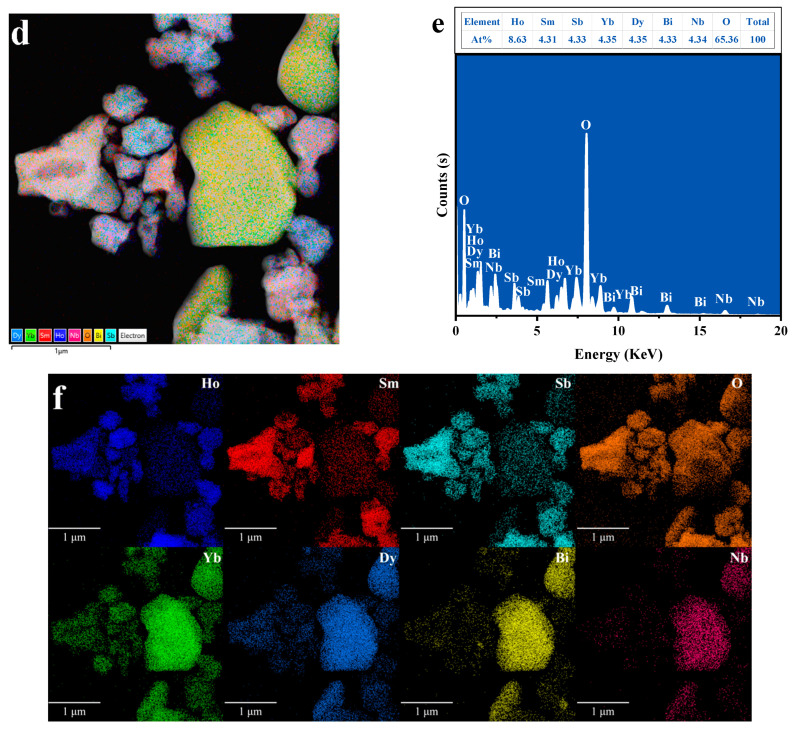
(**a**) TEM, (**b**) SEM, (**c**) HRTEM, (**d**) EDS layered, (**e**) EDS, and (**f**) EDS elemental mapping images of HYO.

The surface features, microstructure, and elemental composition of the synthesized materials were systematically examined using advanced techniques, including transmission electron microscopy (TEM), high-resolution transmission electron microscopy (HRTEM), scanning electron microscopy (SEM), and energy-dispersive X-ray spectroscopy (EDS). Figure 2a,b illustrate the surface features of the individual components, Ho_2_SmSbO_7_ and YbDyBiNbO_7_. Both materials exhibited an irregular block-like shape. Notably, YbDyBiNbO_7_ was uniformly distributed on the surface of Ho_2_SmSbO_7_. The HRTEM image presented in Figure 2c offered valuable insight into the interface between the two components, highlighting its critical role in facilitating efficient charge transfer between the individual constituents. Moreover, the observed lattice fringes, exhibiting a spacing of 0.301 nm, were consistent with the (222) crystallographic plane characteristic of both Ho_2_SmSbO_7_ and YbDyBiNbO_7_ nanoparticles. Elemental mapping, conducted as shown in Figure 2d–f, was used to analyze the distribution of elements within the HYO heterostructure. The EDS spectrum depicted in Figure 2e confirmed the existence of Ho, Sm, Sb, Yb, Dy, Bi, Nb, and O elements evenly distributed across the HYO photocatalyst. The observed elemental distribution revealed that the components within the heterostructure maintain a stoichiometric ratio consistent with those of the individual constituents, Ho_2_SmSbO_7_ and YbDyBiNbO_7_. Moreover, this finding, in conjunction with the results from XRD, FTIR, and Raman analysis, provides compelling evidence for the successful formation of the HYO heterojunction.

**Figure 3:**
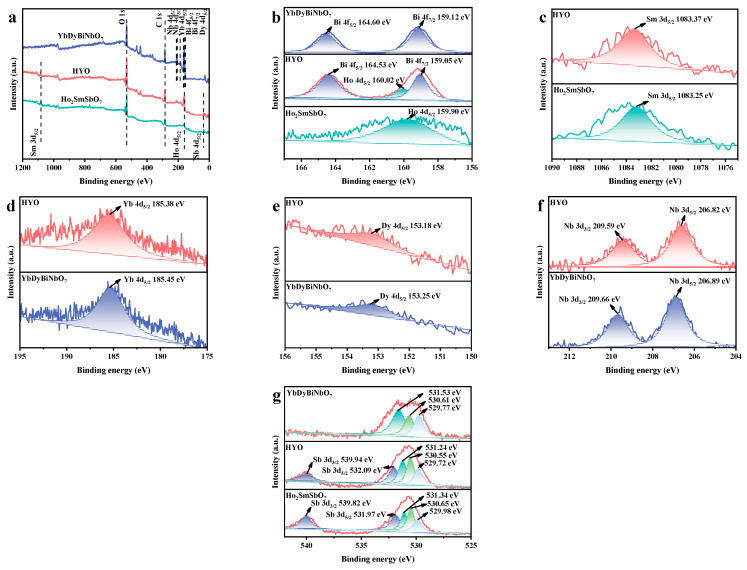
The XPS spectrum of synthesized Ho_2_SmSbO_7_, YbDyBiNbO_7_, and HYO: (**a**) survey spectrum, (**b**–**g**) high-resolution spectra of Bi 4f, Ho 4d, Sm 3d, Yb 4d, Dy 4d, Nb 4d, Sb 3d, and O 1S, respectively.

The analysis of the elements and chemical states of the HYO hybrid photocatalyst was conducted using XPS, with bare Ho_2_SmSbO_7_ and YbDyBiNbO_7_ employed as control samples. The survey spectrum of HYO, depicted in Figure 3a, confirms the presence of Ho, Sm, Sb, Yb, Dy, Bi, Nb, and O elements, indicating their derivation from Ho_2_SmSbO_7_ and YbDyBiNbO_7_. In the Bi 4f region (Figure 3b), the Bi 4f_7/2_ and Bi 4f_5/2_ peaks of YbDyBiNbO_7_ were identified at 159.12 eV and 164.60 eV, respectively, with a spin-orbit splitting of 5.48 eV, consistent with the +3 oxidation state of Bi [71,72,73]. In the HYO composite, these peaks shift slightly to 159.05 eV and 164.53 eV. For Ho, the 4d_5/2_ peak in Ho_2_SmSbO_7_ was located at 159.90 eV (Figure 3c), whereas in HYO, it shifted to 160.02 eV. Similarly, the Sm 3d_5/2_ peak, presented at 1083.25 eV in Ho_2_SmSbO_7_, shifted to 1083.37 eV in HYO. Regarding Yb, the 4d_5/2_ peak of YbDyBiNbO_7_ was detected at 185.45 eV (Figure 3d) and shifted slightly to 185.38 eV in HYO. The Dy 4d_5/2_ peak, observed at 153.25 eV in YbDyBiNbO_7_, also experienced a minor shift to 153.18 eV in HYO (Figure 3e). In the Nb 3d region (Figure 3f), the Nb 3d_3/2_ and Nb 3d_5/2_ peaks of YbDyBiNbO_7_ were positioned at 209.66 eV and 206.89 eV, respectively, with a spin-orbit separation of 2.77 eV, indicative of the +5 oxidation state of Nb [71,72,73]. In HYO, these peaks shifted slightly to 209.59 eV and 206.82 eV, respectively. The Sb 3d region showed the Sb 3d_3/2_ and Sb 3d_5/2_ peaks of Ho_2_SmSbO_7_ at 539.82 eV and 531.97 eV (Figure 3g), shifting to 539.94 eV and 532.09 eV in HYO. The observed positive shifts in binding energies for the Ho 4d, Sm 3d, and Sb 3d peaks in HYO compared to Ho_2_SmSbO_7_, along with the negative shifts in the Yb 4d, Dy 4d, Bi 4f, and Nb 3d peaks relative to YbDyBiNbO_7_, indicated a significant alteration in electron density within the heterojunction [74,75]. Specifically, these shifts suggested an increase in electron density on Yb, Dy, Bi, and Nb while simultaneously reflecting a decrease in electron density on Ho, Sm, and Sb [74,75]. This phenomenon highlighted the successful hybridization of Ho_2_SmSbO_7_ with YbDyBiNbO_7_ and confirmed the formation of the HYO heterojunction. Such findings underscored the intricate electronic interactions within the composite, which were critical for its enhanced functional properties.

Additionally, the O 1s spectra for HYO, Ho_2_SmSbO_7_, and YbDyBiNbO_7_ (Figure 3g) were subjected to deconvolution, revealing three distinct peaks for each material. The peaks identified at 529.72 eV, 529.98 eV, and 529.77 eV are attributable to the presence of lattice oxygen [76]. In contrast, the peaks observed at 530.55 eV, 530.65 eV, and 530.61 eV correspond to adsorbed oxygen species [76]. Additionally, peaks at 531.24 eV, 531.34 eV, and 531.53 eV suggest the presence of surface hydroxyl groups [77].

Elemental surface analysis revealed an average atomic ratio of Ho/Sm/Sb/Yb/Dy/Bi/Nb/O at approximately 861:431:430:433:431:435:433:6546, which closely matched the ratios determined via EDS. The atomic ratios for Ho/Sm/Sb and Yb/Dy/Bi/Nb in the HYO sample were calculated to be 2.00:1.00:1.00 and 1.00:1.00:1.01:1.00, respectively. This concordance supported the successful synthesis of the photocatalysts in alignment with the proposed chemical formula. Notably, the XPS peak analysis of HYO, Ho_2_SmSbO_7_, and YbDyBiNbO_7_ did not indicate the presence of any secondary phases. Collectively, these XPS findings offer critical insights into the development of a Z-scheme heterostructure within the HYO heterojunction and further affirm the strong chemical interactions between Ho_2_SmSbO_7_ and YbDyBiNbO_7_. These results complement and reinforce observations obtained from additional characterization techniques, including XRD, FTIR, Raman Spectroscopy, TEM, SEM, HRTEM, and EDS.

**Figure 4:**
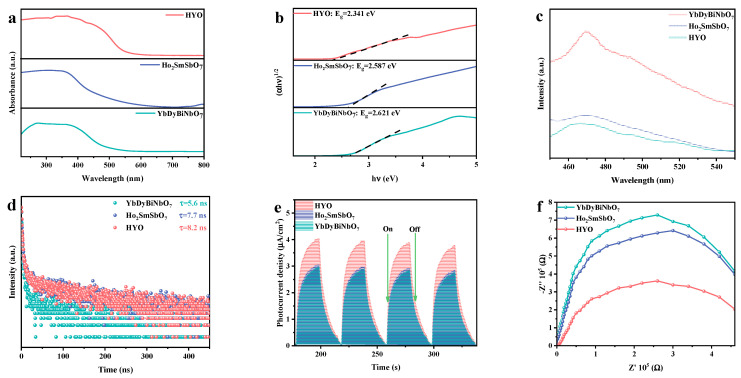
(**a**) UV–vis DRS and (**b**) corresponding plots of (*αhν*)^1/2^ and *hν* for Ho_2_SmSbO_7_, YbDyBiNbO_7_, and HYO; (**c**) PL spectra, (**d**) TRPL spectra, (**e**) PC curves, and (**f**) EIS plots of Ho_2_SmSbO_7_, YbDyBiNbO_7_, and HYO.

As demonstrated in Figure 4a, the diffuse reflectance absorption spectrum was employed to comprehensively analyze the optical absorption characteristics of pure Ho_2_SmSbO_7_, YbDyBiNbO_7_, and HYO. The initiation of absorption for Ho_2_SmSbO_7_ and YbDyBiNbO_7_ was observed at approximately 510 nm and 500 nm, respectively. Whereas, HYO exhibited a notable shift of initiation of absorption at around 560 nm compared to Ho_2_SmSbO_7_ and YbDyBiNbO_7_. The shift suggested that HYO possessed enhanced light absorption capabilities relative to both Ho_2_SmSbO_7_ and YbDyBiNbO_7_.

Figure 4b further detailed the estimated band gap energy values for Ho_2_SmSbO_7_, YbDyBiNbO_7_, and HYO, which were identified as 2.587 eV, 2.621 eV, and 2.341 eV severally, as derived from Equation (1) [78,79,80]:

(1) (αhν)12=A(hν−Eg)

where *A* represents the absorbance factor, *α* denotes the absorption coefficient, *E_g_* is the band gap energy, and *ν* corresponds to the photon energy.

Photoluminescence (PL) measurements were performed to evaluate the recombination rate of PGCs and the photodegradation performance of the synthesized catalysts. Generally, higher PL intensity indicates a greater recombination rate, while lower intensity reflects enhanced transfer efficiency for PGCs [81,82]. As depicted in Figure 4c, broad luminescence peaks were observed around 470 nm, with YbDyBiNbO_7_ exhibiting the highest PL intensity and HYO demonstrating the lowest. This trend indicated that the PGC separation rate in the HYO heterojunction was accelerated, which was advantageous for improving photocatalytic performance.

The time-resolved photoluminescence (TRPL) spectra for Ho_2_SmSbO_7_, YbDyBiNbO_7_, and HYO are presented in Figure 4d. According to Equation (2) [83,84,85],

(2) $$\tau_{ave}=(A_{1}\tau_{1}^{2}+A_{2}\tau_{2}^{2})/(A_{1}\tau_{1}+A_{2}\tau_{2})$$

HYO displayed significantly prolonged lifetimes ($\tau_{ave}$ = 8.2 ns) in comparison to Ho_2_SmSbO_7_ ($\tau_{ave}$ = 7.7 ns) and YbDyBiNbO_7_ ($\tau_{ave}$ = 5.6 ns). The shorter average lifetimes for Ho_2_SmSbO_7_ and YbDyBiNbO_7_ indicated rapid recombination of PGCs, while the longer lifetime for HYO was attributed to efficient separation of PGCs within the HYO heterojunction, corroborating the PL results.

Figure 4e shows the photocurrent (PC) responses recorded for the samples. HYO exhibited a higher photocurrent density compared with pure Ho_2_SmSbO_7_ and YbDyBiNbO_7_, further validating the favorable separation effectiveness of PGCs observed in the PL and TRPL tests [86,87]. Additionally, electrochemical impedance spectroscopy (EIS) Nyquist plots, illustrated in Figure 4f, were utilized to investigate PGC separation behavior. A smaller radius on the Nyquist plot indicates a reduced resistance to charge transfer [88,89]. HYO demonstrated a smaller radius than Ho_2_SmSbO_7_ and YbDyBiNbO_7_, indicating reduced charge transfer resistance and more efficient PGC separation, consistent with previous findings.

The N_2_ adsorption-desorption isotherm curves for the synthesized samples are illustrated in Figure S4. The Brunauer–Emmett–Teller (BET) surface areas for Ho_2_SmSbO_7_, YbDyBiNbO_7_, and HYO were quantified as 1.07, 1.20, and 1.41 m^2^/g, respectively. Importantly, HYO demonstrated a significantly larger specific surface area in comparison to both Ho_2_SmSbO_7_ and YbDyBiNbO_7_. This enhanced surface area correlated with an increased availability of active sites for photodegradation reactions, consequently contributing to an improved photocatalytic efficacy.

### 2.2. Examination of Photocatalytic Efficiency

#### 2.2.1. Photocatalytic Degradation of FNT

**Figure 5:**
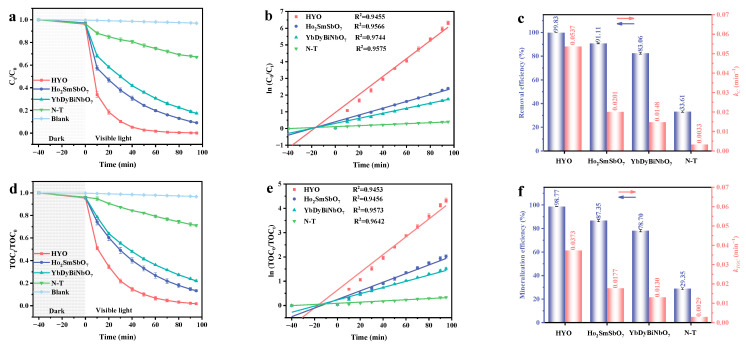
(**a**) Photodegradation, (**b**) kinetic curves, and (**c**) removal efficiencies and kinetic constants for FNT; (**d**) mineralization, (**e**) kinetic curves, and (**f**) mineralization efficiencies and kinetic constants for TOC.

The light-driven catalytic performance for FNT under VE is shown in Figure 5. The solutions were initially agitated in the dark for 40 min to achieve equilibrium between adsorption and desorption. Figure 5a demonstrates that the fabricated catalysts achieved significant FNT degradation efficiencies, with HYO exhibiting notably superior performance compared to Ho_2_SmSbO_7_, YbDyBiNbO_7_, and nitrogen-doped TiO_2_ (N-T). In contrast, control experiments conducted solely with photolysis, omitting the catalysts, revealed no substantial alteration in FNT concentration. This clearly indicated that the observed photodegradation was predominantly attributed to the photocatalytic activity of synthesized catalysts rather than to mere photolytic processes. Figure 5b displays the corresponding ln(*C*_0_/*C*) plots versus irradiation time, which conform to a first-order kinetic model, thus enabling the calculation of kinetic constants for FNT degradation. The standard formula ($ln\frac{C_{0}}{C}=k_{C}t)$ was utilized to calculate the kinetic constants of FNT, where *C* and *C*_0_ represent the concentrations of FNT at interim and initial states, respectively. To elucidate the enhanced photocatalytic performance under VE, Figure 5c summarizes the photodegradation efficiencies (PRE) and the associated rate constants of the samples. The photocatalysts exhibited varying degradation rates in the following sequence: HYO > Ho_2_SmSbO_7_ > YbDyBiNbO_7_ > N-T. Specifically, the PRE of HYO increased by factors of approximately 1.10, 1.20, and 2.97 in comparison with pristine Ho_2_SmSbO_7_, YbDyBiNbO_7_, and N-T, respectively. Additionally, the kinetic constant for HYO demonstrated improvements by factors of approximately 2.67, 3.63, and 16.27 relative to pristine Ho_2_SmSbO_7_, YbDyBiNbO_7_, and N-T. Figure 5d presents the fluctuations in total organic carbon (TOC) saturation observed during the photodecomposition of FNT in agricultural wastewater under VE, employing a range of photocatalysts. Consistent with the results for FNT degradation, the fabricated catalysts achieved substantial TOC mineralization efficiencies. Notably, HYO demonstrated superior performance, achieving mineralization efficiencies that were 1.13 times higher than those of Ho_2_SmSbO_7_, 1.26 times greater than YbDyBiNbO_7_, and an impressive 3.37 times higher than N-T. Figure 5e presents the corresponding ln(*TOC*_0_/*TOC*) plots over time, which also conform to a first-order kinetic model, facilitating the calculation of kinetic constants for TOC degradation. The standard formula ($ln\frac{TOC_{0}}{TOC}=k_{TOC}t)$ was utilized to calculate the kinetic constants of TOC, where *TOC* and *TOC*_0_ represent the concentrations of TOC at interim and initial states, respectively. For enhanced clarity regarding TOC mineralization performance, the mineralization efficiencies and kinetic constants for the samples are depicted in Figure 5f. The photocatalysts exhibited varying mineralization rates, following this order: HYO > Ho_2_SmSbO_7_ > YbDyBiNbO_7_ > N-T. Notably, the kinetic constant for HYO increased by factors of approximately 2.11, 2.87, and 12.86 compared with pristine Ho_2_SmSbO_7_, YbDyBiNbO_7_, and N-T, respectively.

**Figure 6:**
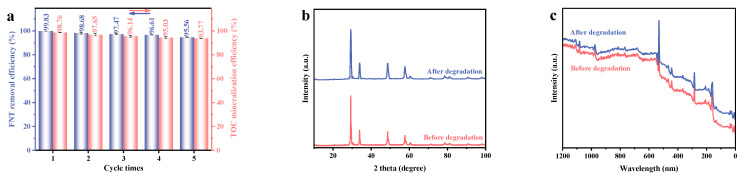
(**a**) Five consecutive tests on HYO for the degradation of FNT and the mineralization of TOC under VE; (**b**) XRD and (**c**) XPS patterns of the fresh and the used HYO.

To evaluate practical durability and reusability, recirculation evaluations were conducted with the synthesized HYO, the results of which were shown in Figures S5 and Figure 6a. Remarkably, HYO demonstrated the ability to retain more than 95% of its degradation efficiencies and more than 93% of its mineralization efficiencies following five consecutive photodecomposition processes, indicating its potential effectiveness as a photocatalyst for the removal of FNT contaminants in wastewater remediation applications. To visually evaluate the structural stability of HYO, XRD and XPS analyses were performed on fresh and used samples. As illustrated in Figure 6b,c, the characteristic peaks of the used HYO closely aligned with those of the fresh samples, and no extra diffraction peaks were detected in the XRD or XPS patterns of HYO after undergoing successive photocatalytic degradation. The characterization findings indicate that the heterojunction sample demonstrates impressive stability in both its physical and chemical properties.

**Figure 7:**
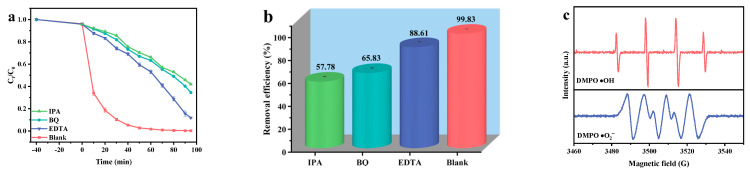
Impact of different radical scavengers on (**a**) FNT saturation, (**b**) removal efficiency of FNT, and (**c**) EPR spectrum for DMPO·O_2_^−^ and DMPO·OH over HYO.

To elucidate the active species generated by HYO during the photodegradation of FNT, a series of radical scavenging experiments were performed. The incorporation of benzoquinone (BQ), isopropanol (IPA), and ethylene diamine tetraacetic acid (EDTA) functioned as selective scavengers targeting •O_2_^−^, •OH, and h^+^, respectively. The results in Figure 7a,b demonstrated a significant decrease in removal efficiencies, which varied from 99.83% in the absence of scavengers to approximately 57.78%, 65.83%, and 88.16% upon the addition of IPA, BQ, and EDTA, respectively. These findings indicate that •OH is critical to the photocatalytic degradation process. Electron paramagnetic resonance (EPR) measurements were utilized to provide additional confirmation regarding the active species produced by HYO. As depicted in Figure 7c, distinct signals corresponding to DMPO•OH adducts were observed, displaying a quartet peak ratio of 1:2:2:1, indicative of hydroxyl radical formation [90]. Additionally, a six-peak signal associated with DMPO•O_2_^−^ was clearly detected [90]. Importantly, the intensity profiles of the EPR signals suggested a predominance of •OH radicals over •O_2_^−^ radicals in the system [90,91]. Furthermore, EPR measurements were conducted to confirm the active species generated by Ho_2_SmSbO_7_ and YbDyBiNbO_7_. As shown in Figure S6, distinctive signals corresponding to DMPO•OH adducts were observed in the case of Ho_2_SmSbO_7_, demonstrating a quartet peak ratio of 1:2:2:1, indicative of hydroxyl radical formation. In contrast, no DMPO•O_2_^−^ signal was recorded for this compound. Figure S7 illustrates that a pronounced six-peak signal associated with DMPO•O_2_^−^ was observed in YbDyBiNbO_7_, while no DMPO•OH signal was detected. The EPR results, in conjunction with the findings from the radical scavenger experiments, provide compelling evidence for the establishment of the photocatalytic mechanism of HYO. Additionally, these combined analyses confirm the crucial roles of both •OH and •O_2_^−^ radicals in the photocatalytic degradation process, further elucidating the active species involved in the photodegradation of FNT.

#### 2.2.2. Possible Photocatalytic Mechanism of HYO

**Figure 8:**
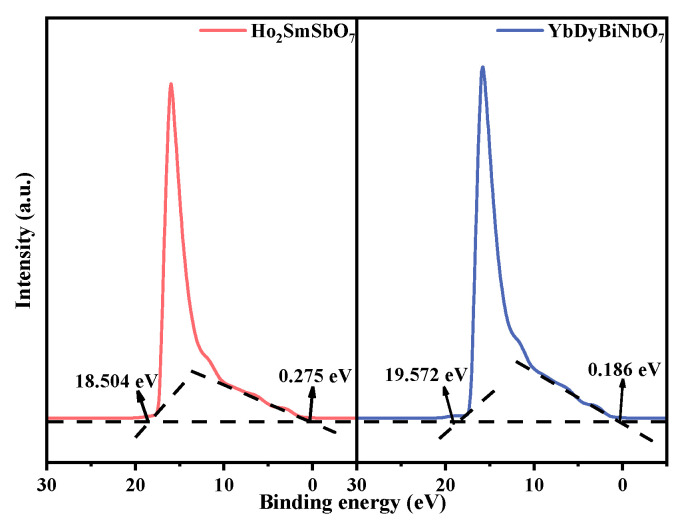
UPS spectra of Ho_2_SmSbO_7_ and YbDyBiNbO_7_ (the intersections of the black dash lines indicated by the black arrows indicated the onset (Ei) and cutoff (Ecutoff) binding energy).

To assess the ionization potentials of the individual components of HYO, ultraviolet photoelectron spectroscopy (UPS) was employed. The spectrum presented in Figure 8 indicated that the onset and cutoff binding energies for Ho_2_SmSbO_7_ were 0.275 eV and 18.504 eV, respectively, while for YbDyBiNbO_7_, these values were 0.186 eV and 19.572 eV [92]. Taking into account the excitation energy of approximately 21.2 eV, the ionization potentials for Ho_2_SmSbO_7_ and YbDyBiNbO_7_ were determined to be 2.971 eV and 1.864 eV severally [93,94]. Utilizing ionization potentials, the conduction band (CB) potentials for Ho_2_SmSbO_7_ and YbDyBiNbO_7_ were estimated to be 0.384 eV and −0.757 eV severally.

**Figure 9:**
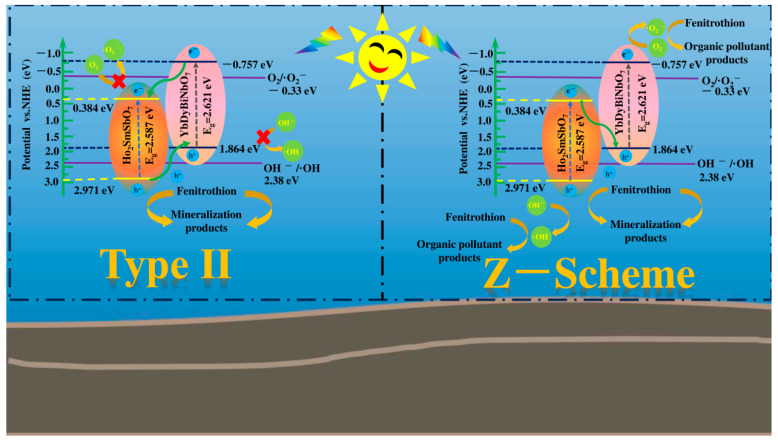
Plausible photodegradation mechanism of FNT with HYO as photocatalyst under VE.

Drawing on the analyses conducted earlier, the potential charge transfer pathways within the HYO heterojunction were depicted in Figure 9. Considering the CB and valence band (VB) positions of Ho_2_SmSbO_7_ and YbDyBiNbO_7_, two plausible photocatalytic mechanisms, Z-scheme and Type II, could be proposed for HYO. In the Z-scheme configuration, in response to visible light stimulation, photogenerated electrons were predicted to move from the CB of Ho_2_SmSbO_7_ to the VB of YbDyBiNbO_7_. This electron transfer facilitated effective charge separation while maintaining elevated reduction and oxidation potentials conducive to photocatalytic reactions. Under these conditions, the h^+^ in the VB of Ho_2_SmSbO_7_ was capable of reacting with OH^−^ to form •OH, which subsequently decomposes the target contaminant. Concurrently, the electrons in the CB of YbDyBiNbO_7_ could react with O_2_, leading to the generation of •O_2_^−^. These analytic results were consistent with the experimental results obtained from radical trapping experiments and EPR analyses, which detected both •O_2_^−^ and •OH radicals in HYO heterojunction. Conversely, the Type II mechanism posited that photogenerated electrons accumulated in the CB of Ho_2_SmSbO_7_, which had a reduced reduction potential, and in the VB of YbDyBiNbO_7_, characterized by a reduced oxidation potential. The CB potential of Ho_2_SmSbO_7_ was positioned at a higher potential than the O_2_/•O_2_^−^ (−0.33 eV vs. NHE) [95], indicating that electrons residing in the CB of Ho_2_SmSbO_7_ were found to be ineffective in reducing O_2_ to generate •O_2_^−^. Additionally, the formation of •OH in the VB of YbDyBiNbO_7_ was likely to be impaired due to the negative VB potential, which was lower than the OH^−^/•OH reduction potential (2.38 eV vs. NHE) [96,97]. This limitation undermined degradation reactions and contradicted the experimental detection results of •O_2_^−^ and •OH radicals observed in trapping experiments and EPR analyses of HYO heterojunction.

Consequently, the findings indicate that under VE, the charge migration pathway in the heterojunction composed of Ho_2_SmSbO_7_ and YbDyBiNbO_7_ primarily followed the Z-scheme mechanism, which is particularly advantageous for enhancing the efficiency of the photocatalytic process. The markedly improved photocatalytic performance of HYO could be attributed to the successful establishment of a Z-scheme heterojunction between Ho_2_SmSbO_7_ and YbDyBiNbO_7_.

Additionally, in the context of photocatalytic carbon dioxide (CO_2_) reduction, the minimum reduction potentials required to convert CO_2_ into carbon monoxide (CO) and methane (CH_4_) are −0.51 eV and −0.24 eV (vs. NHE), respectively. Consequently, within the Z-scheme HYO heterojunction, photogenerated electrons in the CB of YbDyBiNbO_7_ could effectively facilitate the reduction of carbon dioxide to CO and CH_4_ upon VE, as depicted in Figure S8. This observation highlights the significant potential of HYO heterojunction photocatalyst in advancing the field of photocatalytic CO_2_ reduction.

#### 2.2.3. Possible Degradation Pathway of FNT

**Figure 10:**
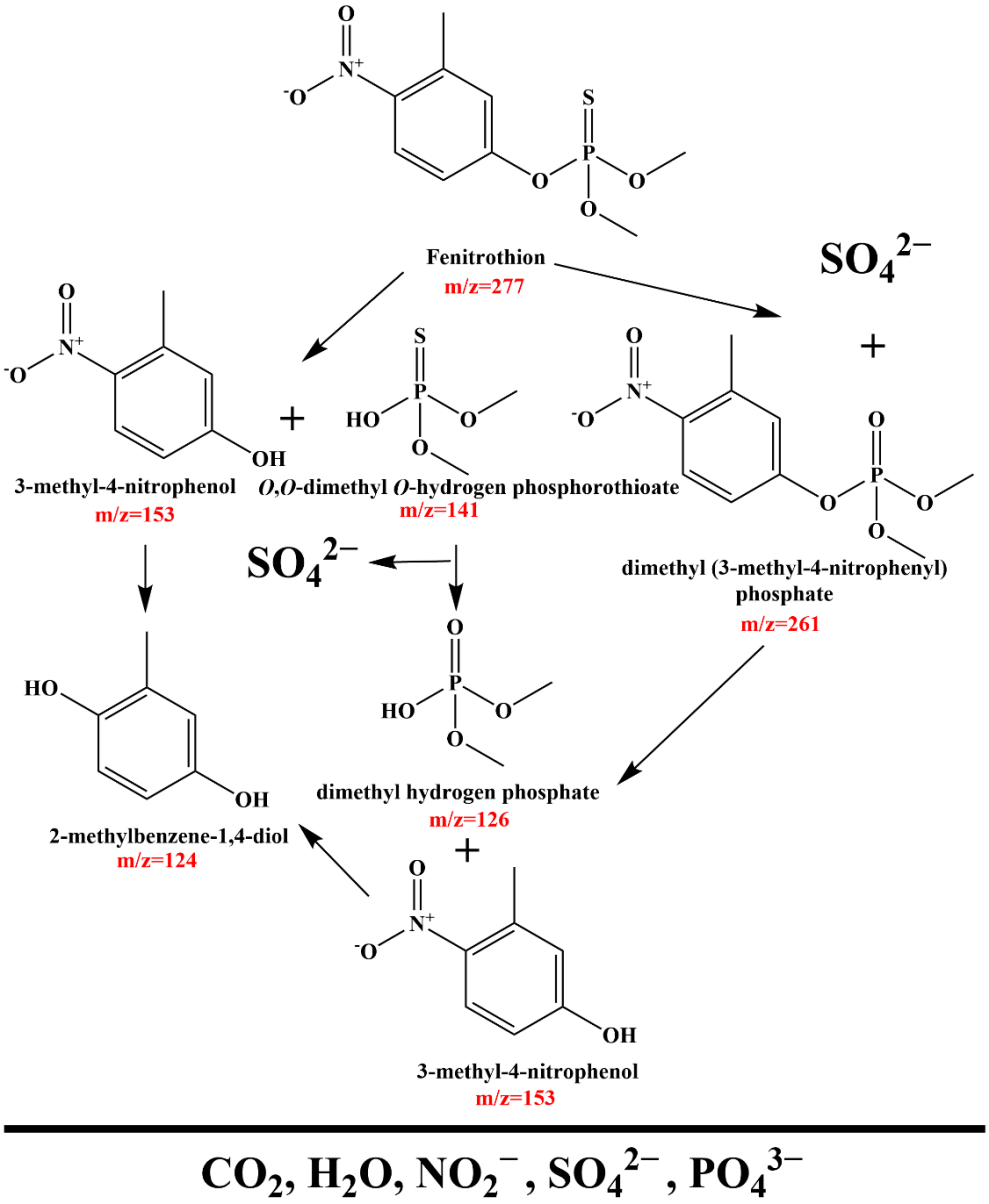
Viable photodegradation pathway for FNT under VE with HYO heterojunction as catalyst.

Drawing upon the existing literature and results obtained from liquid chromatography-mass spectrometry (LC-MS) experiments, potential degradation pathways for FNT (*m*/*z* = 277) have been proposed, as illustrated in Figure 10 [5,98,99]. The analysis identified two distinct degradation mechanisms. The first pathway involved the cleavage of the ester bond between the dimethyl ester of O,O-dimethyl O-hydrogen phosphorothioate (*m*/*z* = 141) and 3-methyl-4-nitrophenol (*m*/*z* = 153) [98]. This pathway signified the initial breakdown of the molecule into simpler constituents. In the second pathway, a sulfur atom was substituted by an oxygen atom, leading to the formation of dimethyl (3-methyl-4-nitrophenyl) phosphate (*m*/*z* = 261) and sulfate ions [98,99]. Following this sulfur substitution, this pathway paralleled the first, where cleavage of the ester bond occurred between dimethyl hydrogen phosphate (*m*/*z* = 126) and 3-methyl-4-nitrophenol [98]. Further transformation of the phenolic moiety was noted, where successive substitutions of the nitro (NO_2_) grouped on the aromatic ring yield 2-methylbenzene-1,4-diol (*m*/*z* = 124) [98]. Ultimately, both dimethyl hydrogen phosphate and 2-methylbenzene-1,4-diol were further degraded into carbon dioxide (CO_2_), water (H_2_O), and inorganic anions such as sulfate (SO_4_^2−^), phosphate (PO_4_^2−^), and nitrite (NO_2_^−^).

## 3. Experimental Section

### 3.1. Materials and Reagents

All chemicals were employed in the as-received forms without any further purification. The following reagents were sourced from Macklin Biochemical Co., Ltd., Shanghai, China: Sm(NO_3_)_3_·6H_2_O (purity = 99.99%), Ho(NO_3_)_3_·5H_2_O (purity = 99.99%), NbCl_5_ (purity = 99.999%), SbCl_5_ (purity = 99.999%), Dy(NO_3_)_3_·5H_2_O (purity = 99.99%), and benzoquinone (BQ, C_6_H_4_O_2_, purity ≥ 99.5%). Additionally, Bi(NO_3_)_3_·5H_2_O (purity = 99.999%), ethylenediaminetetraacetic acid (EDTA, C_10_H_16_N_2_O_8_, purity = 99.995%), and pure ethanol (C_2_H_5_OH, purity ≥ 99.5%) were obtained from Merck Co., Ltd., Shanghai, China. Furthermore, Yb(NO_3_)_3_·5H_2_O (purity ≥ 99.999%), octanol (C_8_H_18_O, purity ≥ 99.5%), fenitrothion (FNT, C_9_H_12_NO_5_PS, analytical standard), and isopropyl alcohol (IPA, C_3_H_8_O, purity ≥ 99.999%) were procured from Aladdin Group Chemical Reagent Co., Ltd., Shanghai, China.

### 3.2. Synthesis of N-Doped TiO_2_

A specified quantity of tetrabutyl titanate was combined with absolute ethanol to produce Solution A. Under the conditions of magnetic stirring, Solution A was introduced dropwise into Solution B, which consisted of a mixture of glacial acetic acid, double-distilled water, and absolute ethanol. Following 30 min of vigorous stirring of the combined Solutions A and B, varying quantities of 1 mol/L ammonia solution, maintaining a molar ratio of N/Ti at 8%, were incrementally added to the mixture. The stirring process was then continued for an additional 120 min. The resultant mixture was allowed to equilibrate at room temperature for 1 day. Subsequently, it was transferred to a drying oven where it was subjected to heat at 105 °C for a duration of 4 h. The solid was taken out and ground, and then the powder obtained by calcining at 400 °C for 2 h in a high-temperature furnace was ground again to obtain nitrogen-doped titanium dioxide powder.

### 3.3. Preparation Method of Ho_2_SmSbO_7_

In this study, the Ho_2_SmSbO_7_ catalyst was synthesized using a solvothermal method. Initially, equal volumes of precursor solution, Ho(NO_3_)_3_·5H_2_O (0.42 mol/L), Sm(NO_3_)_3_·6H_2_O (0.21 mol/L), and SbCl_5_ (0.21 mol/L) were thoroughly mixed and subjected to magnetic stirring for 1350 min. The homogeneous precursor mixture obtained was subsequently placed into a high-pressure autoclave lined with PTFE, where it was subjected to a temperature of 200 °C for a duration of 850 min. Subsequently, the mixture was subjected to heating in a tube furnace under a nitrogen atmosphere at a rate of 5 °C/min until it attained a temperature of 300 °C, which was then maintained for an additional 600 min. This procedure led to the successful synthesis of pure Ho_2_SmSbO_7_ powder.

### 3.4. Preparation Method of YbDyBiNbO_7_

The solvothermal method was likewise utilized for the synthesis of the photocatalytic material YbDyBiNbO7. Equal volumes of precursor solutions—Yb(NO_3_)_3_·5H_2_O (0.21 mol/L), Dy(NO_3_)_3_·5H_2_O (0.21 mol/L), Bi(NO_3_)_3_·5H_2_O (0.21 mol/L), and NbCl_5_ (0.21 mol/L)—were combined. Following thorough mixing and magnetic stirring for 1500 min, the precursor solution was placed into an autoclave, where it was heated to 220 °C and held at the temperature for 1150 min. Subsequently, the mixture was heated in a tube furnace at a rate of 8 °C/min until it attained a final temperature of 800 °C, at which point it was maintained for 1200 min. This process effectively resulted in the production of pure YbDyBiNbO_7_ powder.

### 3.5. Synthesis of HYO Heterojunction Photocatalyst

HYO was synthesized through an ultrasound-assisted solvothermal approach. An equal amount of Ho_2_SmSbO_7_ and YbDyBiNbO_7_, which were previously obtained by solvothermal synthesis, were combined in octanol and subjected to sonication for 300 min in an ultrasonic bath. Following this, the mixture underwent magnetic stirring while being maintained at 190 °C for 200 min to promote the deposition of Ho_2_SmSbO_7_ onto the surface of YbDyBiNbO_7_ nanoparticles, leading to the formation of the HYO heterostructure catalyst. Upon reaching room temperature, the resultant product was isolated through centrifugation and subsequently subjected to several ethanol washes to ensure thorough purification. The purified powder was subjected to drying in a vacuum oven at a temperature of 110 °C for 90 min and subsequently stored in a desiccator for later application. As a result, the HYO heterostructure catalyst was successfully synthesized.

### 3.6. Characterization

The descriptions of the characterization details are reported in the Supplementary Materials (Section S1).

### 3.7. Photoelectrochemical Experiments

To prepare the photocatalytic material for electrochemical characterization, 3 mg of the synthesized photocatalyst was initially suspended in a solvent mixture consisting of 300 µL of ethanol and ethylene glycol. The suspension was then subjected to ultrasonic treatment for a duration of 30 min to achieve a uniform dispersion of the particles. Following sonication, the mixture was vigorously stirred for 2 h to achieve a uniform suspension. Subsequently, 30 µL of the prepared suspension was evenly deposited onto a glassy carbon electrode and permitted to air-dry for 30 min. In the electrochemical measurements, a platinum plate was employed as the counter electrode, with an Ag/AgCl electrode functioning as the reference electrode. The transient photocurrent response curves of the samples were acquired using a CHI-660D electrochemical workstation (Chenhua Instruments Co., Ltd., Shanghai, China), utilizing a 0.12 mol·L^−1^ Na_2_SO_4_ solution as the electrolyte. Additionally, EIS was conducted in a 0.12 mol·L^−1^ KCl solution to further assess the electrochemical properties of the photocatalytic material.

### 3.8. Experimental Setup and Procedure

A concentration of 0.4 g/L of photocatalysts (Ho_2_SmSbO_7_, YbDyBiNbO_7_, and HYO) was introduced into a 480 mL solution of FNT at 10 mg/L to assess their adsorption and photodegradation activities. The experiments were carried out in a photocatalytic reactor (CEL-LB70, China Education Au-Light Technology Co., Ltd., Beijing, China). To promote uniform dispersion of the catalysts throughout the photocatalytic reaction platform and achieve saturation of FNT adsorption, initial adsorption was conducted in the dark for a duration of 40 min.

The subsequent photodegradation process was initiated under VE using a 500 W Xenon lamp equipped with a 420 nm filter. Throughout the photodegradation process, 5 mL samples of the reacted suspension were collected at 10 min intervals. Each sample was subsequently subjected to centrifugation at 7200 rpm for 10 min to separate the clear liquid, which was then used for further analytical investigations. Residual FNT concentration was measured employing an Agilent 200 high-performance liquid chromatography (HPLC) system (Agilent Technologies, Palo Alto, CA, USA). A 10 µL volume of the clear supernatant was injected into the HPLC at a flow rate of 0.5 mL/min for analysis.

To investigate the mineralization of TOC during the degradation of FNT, a TOC analysis was performed utilizing a TOC analyzer (TOC-5000 A, Shimadzu Corporation, Kyoto, Japan). Potassium acid phthalate (KHC_8_H_4_O_4_) WAS employed as standard reference material for calibrating TOC measurements throughout the photodecomposition of FNT. Calibration standards were established with carbon concentrations spanning from 0 to 100 mg/L, utilizing potassium acid phthalate as the reference standard for calibration.

Additionally, for the characterization of intermediate reaction products, a Thermo Quest LCQ Duo liquid chromatography-mass spectrometry (LC-MS) system (Thermo Fisher Scientific Corporation, Waltham, MA, USA) was employed with a Beta Basic-C18 HPLC column. Upon completion of the photocatalytic reaction, an automatic injection of a 20 µL aliquot from the resultant solution was performed into the LC-MS system. The mobile phase utilized for the analysis comprised a mixture of 60% methanol and 40% ultrapure water, while the mass spectrometry settings were calibrated to detect a mass-to-charge ratio (*m*/*z*) range from 50 to 500.

## 4. Conclusions

In summary, a direct Z-scheme heterojunction photocatalyst, HYO, was successfully synthesized using a straightforward ultrasonic-assisted solvent thermal method. The findings indicate a distinct Z-scheme charge transfer mechanism between Ho_2_SmSbO_7_ and YbDyBiNbO_7_, significantly enhancing the separation efficiency of PGCs. Furthermore, the formation of this Z-scheme heterojunction markedly improves the redox capabilities of HYO. Photoelectrochemical tests demonstrated that the optimized HYO materials exhibited remarkable efficiencies in separating PGCs. In experiments utilizing FNT as a model pollutant, HYO displayed extraordinary photodegradation activity under visible light, achieving degradation rates that were 1.10, 1.20, and 2.97 times greater than those of pure Ho_2_SmSbO_7_, YbDyBiNbO_7_, and N-T, respectively. Moreover, the stability of the HYO heterojunction was substantiated through cyclic experiments and complementary characterizations. Quenching experiments and EPR analyses revealed the pivotal roles of •O_2_^−^, •OH, and h^+^ in the photocatalytic degradation of FNT. Additionally, a credible degradation pathway and mechanism for FNT degradation, along with a potential mechanism for CO_2_ reduction, were proposed. In conclusion, the HYO heterojunction demonstrates significant potential for applications in the remediation of pesticide-contaminated wastewater and photocatalytic CO_2_ reduction.

## Data Availability

Data are contained within the article.

## Supplementary Materials

The following supporting information can be downloaded at: https://www.mdpi.com/article/10.3390/molecules29245930/s1, Figure S1: XRD pattern of Ho_2_SmSbO_7_ and YbDyBiNbO_7_,and standard card of Bi_2_InNbO_7_; Figure S2: (a) XRD pattern and Rietveld refinement and (b) the atomic architecture (red atom: O; green atom: Ho; purple atom: Sm or Sb) of Ho_2_SmSbO_7_; Figure S3: (a) XRD pattern and Rietveld refinement and (b) the atomic architecture (red atom: O; green atom: Yb or Dy; cyan atom: Bi or Nb) of YbDyBiNbO_7_; Figure S4: The N_2_ sorption isotherms of of Ho_2_SmSbO_7_, YbDyBiNbO_7_, and HYO; Figure S5: (a) Photodegradation; (b) kinetic curves, and (c) removal efficiencies and kinetic constants for five consecutive FNT degradation tests; (d) mineralization, (e) kinetic curves, and (f) mineralization efficiencies and kinetic constants for five consecutive TOC mineralization tests; Figure S6: EPR spectrum for DMPO·OH and DMPO·O_2_^−^ over H_2_SmSbO_7_; Figure S7: EPR spectrum for DMPO·O_2_^−^ and DMPO·OH over YbDyBiNbO_7_; Figure S8: Plausible photocatalytic CO_2_ reduction mechanism of HYO under VE; Table S1: Configurable properties of Ho_2_SmSbO_7_ fabricated using solvothermal method; Table S2: Configurable properties of YbDyBiNbO_7_ fabricated using solvothermal method; Section S1: Characterization.
