# Supplementary material for: Visible Light-Driven Direct Z-Scheme Ho2SmSbO7/YbDyBiNbO7 Heterojunction Photocatalyst for Efficient Degradation of Fenitrothion
Source: Molecules. 2024 Dec 16;29(24):5930. doi: 10.3390/molecules29245930 (PMC11678090; doi:10.3390/molecules29245930)
Supplement: Supplementary file 1 [file molecules-29-05930-s001.zip › molecules-3314646-supplementary.pdf]

Visible Light-Driven Direct Z-Scheme Ho_2SmSbO_7/$YbDyBiNbO_7$ Heterojunction Photocatalyst for Efficient Degradation of Fenitrothion

Liang Hao [1] and Jingfei Luan [1,2,*]

[1] School of Physics, Changchun Normal University, Changchun 130032, China; hliang0725@163.com
[2] State Key Laboratory of Pollution Control and Resource Reuse, School of the Environment, Nanjing University, Nanjing 210093, China
* Correspondence: jfluan@nju.edu.cn; Tel.: +86-19951939498

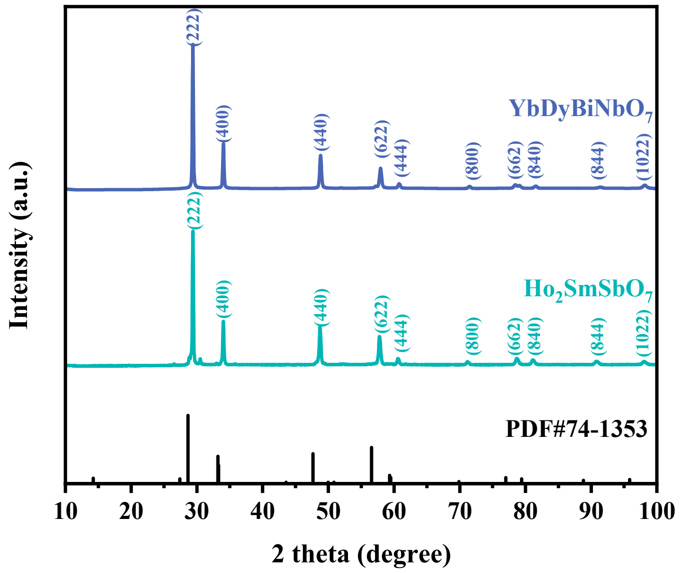

Figure S1. XRD pattern of Ho_2SmSbO_7 and $YbDyBiNbO_7$,and standard card of Bi2InNbO7.

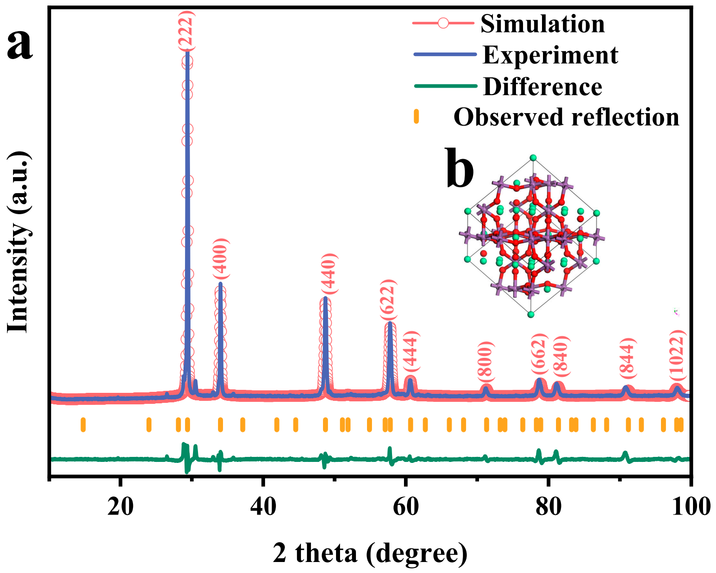

Figure S2. (**a**) XRD pattern and Rietveld refinement and (**b**) the atomic architecture (red atom: O; green atom: Ho; purple atom: Sm or Sb) of Ho_2SmSbO_7.

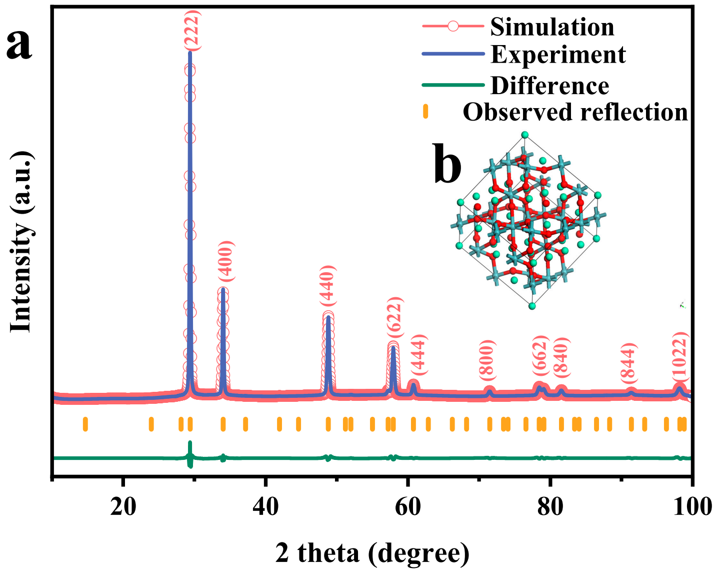

Figure S3. (**a**) XRD pattern and Rietveld refinement and (**b**) the atomic architecture (red atom: O; green atom: Yb or Dy; cyan atom: Bi or Nb) of $YbDyBiNbO_7$.

Table S1. Configurable properties of Ho_2SmSbO_7 fabricated using solvothermal method.

Atom	x	y	z	Occupation Factor
Ho	0	0	0	1
Sm	0.5	0.5	0.5	0.5
Sb	0.5	0.5	0.5	0.5
O(1)	−0.178	0.125	0.125	1
O(2)	0.125	0.125	0.125	1

Table S2. Configurable properties of $YbDyBiNbO_7$ fabricated using solvothermal method.

Atom	x	y	z	Occupation Factor
Yb	0	0	0	0.5
Dy	0	0	0	0.5
Bi	0.5	0.5	0.5	0.5
Nb	0.5	0.5	0.5	0.5
O(1)	−0.178	0.125	0.125	1
O(2)	0.125	0.125	0.125	1

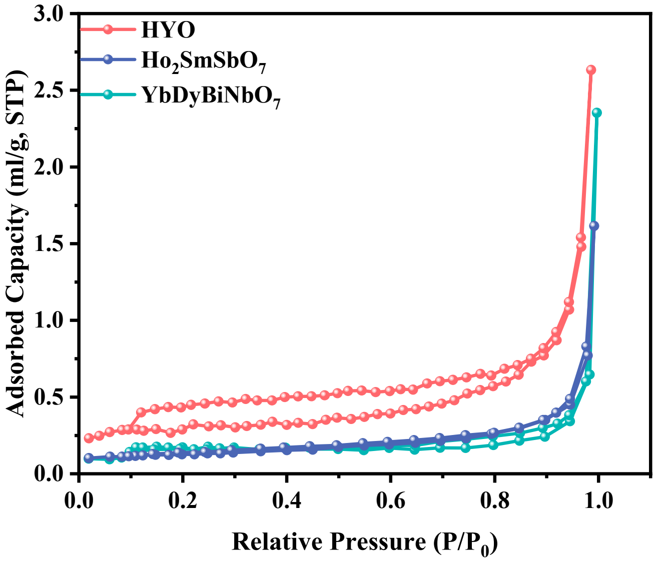

Figure S4. The N_2 sorption isotherms of of Ho_2SmSbO_7, $YbDyBiNbO_7$, and HYO.

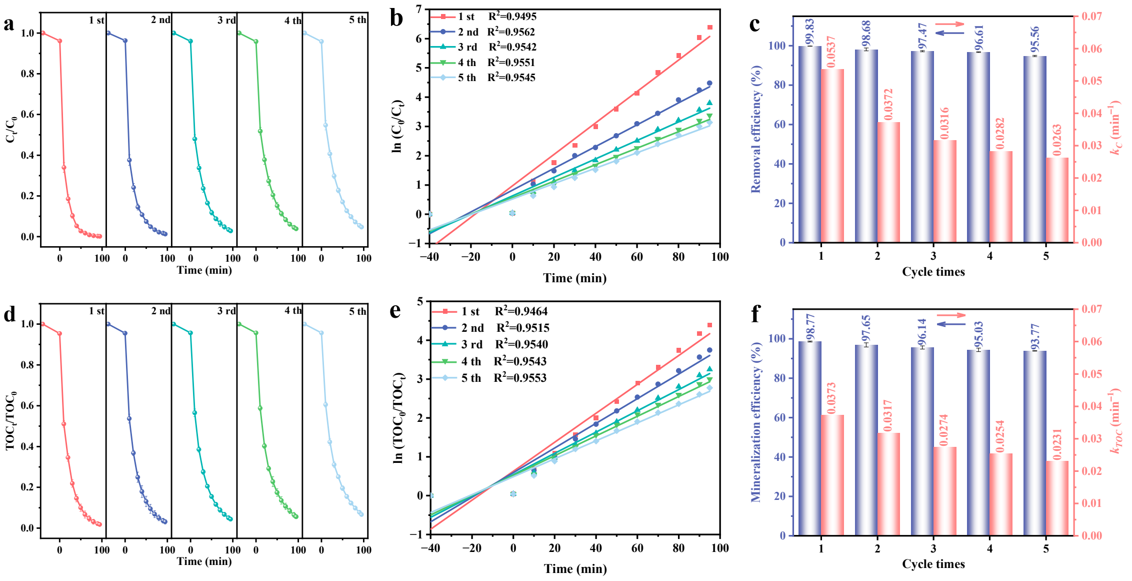

Figure S5. (a) Photodegradation; **(b)** kinetic curves, and **(c)** removal efficiencies and kinetic constants for five consecutive FNT degradation tests; **(d)** mineralization, **(e)** kinetic curves, and **(f)** mineralization efficiencies and kinetic constants for five consecutive TOC mineralization tests.

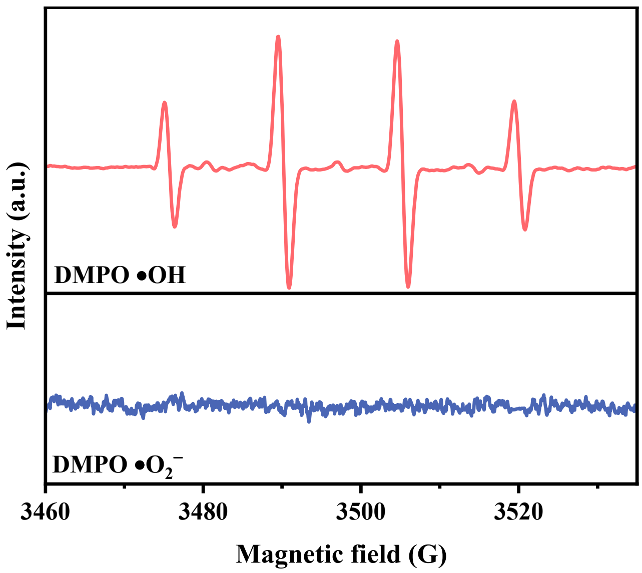

Figure S6. EPR spectrum for DMPO·OH and DMPO·O_2^- over H_2SmSbO_7.

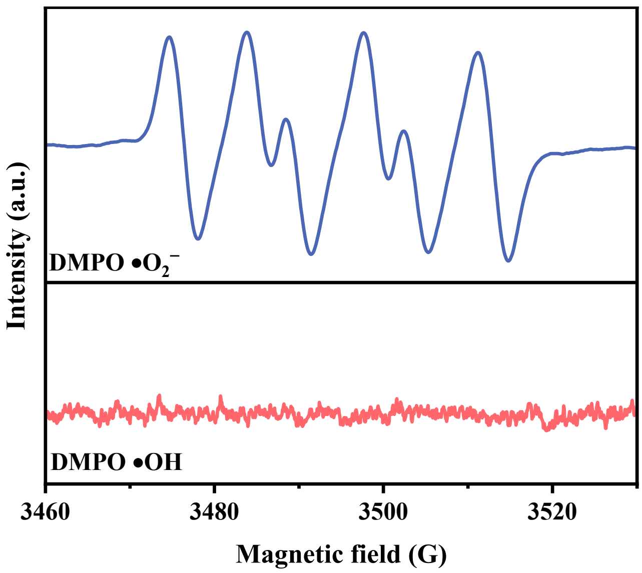

Figure S7. EPR spectrum for DMPO·O_2^- and DMPO·OH over $YbDyBiNbO_7$.

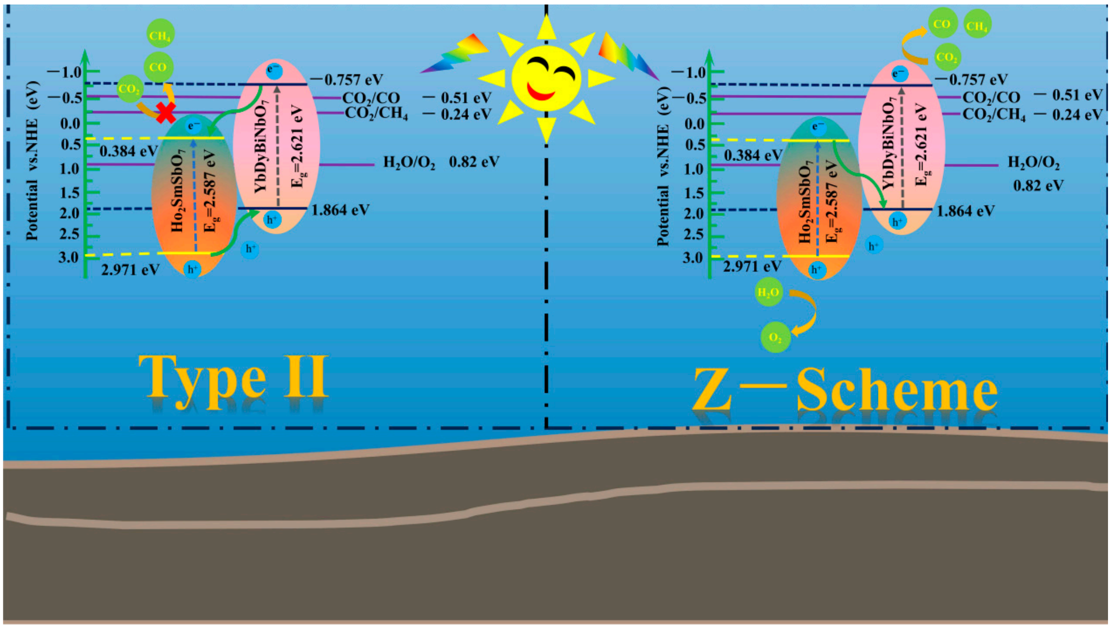

Figure S8. Plausible photocatalytic CO2 reduction mechanism of HYO under VE.

Section S1. Characterization

Crystallographic data were acquired through X-ray diffraction (XRD) utilizing a Shimadzu XRD-6000 diffractometer, based in Kyoto, Japan. Functional groups and chemical bonding were analyzed using Fourier-transform infrared spectroscopy (FTIR) with a WQF-530A spectrometer from Beifen-Ruili Analytical Instrument (Group) Co., Ltd. in Beijing, China. The interactions of chemical bonds were further examined through Raman spectroscopy using an INVIA0919-06 system provided by RENSHAW plx, based in Wotton-under-Edge, Gloucestershire, UK. The microstructural and morphological characteristics were investigated via transmission electron microscopy (TEM) with a Talos F200X G2 instrument from Thermo Fisher Scientific, located in Waltham, MA, USA. Surface topography was analyzed through scanning electron microscopy (SEM), utilizing a SU8010 model from Hitachi, based in Kyoto, Japan. Elemental analysis was carried out employing energy-dispersive spectroscopy (EDS). X-ray photoelectron spectroscopy (XPS) was conducted using a PHI 5000 VersaProbe instrument from UlVAC-PHI in Maoqi City, Japan, to analyze the surface chemical composition and oxidation states. Optical properties of the samples were assessed using ultraviolet-visible diffuse reflectance spectrophotometry (UV-Vis DRS) with a UV-3600 spectrophotometer also from Shimadzu Corporation. Additionally, the properties of photoelectrochemical were characterized with an FLS980 spectrophotometer from Edinburgh Instruments Ltd. in Edinburgh, UK. The specific surface area of the samples were determined with BSD-PM2 specific surface area and porosity analyzer from BEISHIDE Instruments Technology (Beijing) Co., Ltd. in Beijing, China. Lastly, electron paramagnetic resonance (EPR) spectroscopy was employed to detect free radicals in the samples, utilizing an A300 instrument from Bruker Corporation in Karlsruhe, Germany. Ultraviolet photoelectron spectroscopy (UPS) was performed to measure the ionization potential of the valence band with an Escalab 250 xi instrument from Thermo Fisher Scientific in Waltham, MA, USA.
